# Evaluation of the remineralizing effect of biomimetic self-assembling peptides in post-orthodontic white spot lesions compared to fluoride-based delivery systems: randomized controlled trial

**DOI:** 10.1007/s00784-022-04757-7

**Published:** 2022-10-26

**Authors:** Raneen Ahmed Abou El Gheit Gohar, Shereen Hafez Ibrahim, Omaima Mohamed Safwat

**Affiliations:** 1 Department of Conservative Dentistry, Faculty of Dentistry, Cairo University, 11 EL-Saraya St. Manial, Cairo, 11553 Egypt; 2 Faculty of Dentistry, Suez University, Suez, Egypt

**Keywords:** Self-assembling peptides, SAP, Fluoride-based delivery systems, Post-orthodontic white spot lesions, Remineralization, Biomimetic

## Abstract

**Objectives:**

To evaluate the clinical performance of self-assembling peptides versus fluoride-based delivery systems in post-orthodontic white spot lesions.

**Materials and methods:**

The participants were randomly assigned into two groups (*n* = 58) according to the remineralizing agent used, where (A) group represented participants receiving a varnish containing 22.600 fluoride ppm and tricalcium phosphate, while the second group (B) represented participants receiving self-assembling peptide. The remineralizing process of the white spot lesion was assessed using the DIAGNOdent pen and ICDAS scoring system according to the time when the remineralizing agent was used (T), where T0 represented the score taken at baseline. T1 represented the score taken after 3 months of follow-ups and T2 score represented the score taken after 6 months of follow-up. Data were collected and statistically analyzed. The parametric data: two-way ANOVA was used to test the effect of interaction among different variables. The non-parametric data: Mann–Whitney test was used. The significance level was set at *p* ≤ 0.05.

**Results:**

There was a quantitative statistically significant difference via DIAGNOpen readings between Group A (fluoride material) and Group B (self-assembling peptides). The highest mean value of 10.51 was found in Group A, while the least mean value of 6.45 was found in Group B. Besides, there was a significant difference in each group concerning the time factors T0, T1, and T2 groups where (*p* < 0.001. As for the qualitative results concerning the ICDAS score, there was no significant difference between the two groups along with the follow-up periods T0, T1, and T2 where the *p* value is equal to 0.064, 0.087, and 0.277 respectively.

**Conclusions:**

The visual assessment using ICDAS reveals that the biomimetic remineralization using self-assembling peptides and the fluoride-based varnish material showed a similar effect in masking post-orthodontic white spot lesions. However, the laser fluorescence using DIAGNOpen showed that the self-assembling peptides reveal higher performance in subsurface remineralization than the fluoride-based varnish material. Therefore, self-assembling peptides are considered a promising material for lesion regression in post-orthodontics white spot lesions.

**Clinical relevance:**

Self-assembling peptide SAP-14 is a new approach to reverse and mask off post-orthodontics white spot lesions.

**Supplementary Information:**

The online version contains supplementary material available at 10.1007/s00784-022-04757-7.

## Introduction

Dental caries is one of the most prevalent chronic infectious diseases, which remains a major public health concern. Dental caries passes through multi-stages, starting from molecular changes in the apatite crystal to visible white spot lesions, through to dentin involvement, and eventual cavitation. Advancement through these phases requires continual imbalance between pathological and protecting factors, resulting in the dissolution of crystals apatite and a net loss of calcium and phosphate which is known as demineralization [1, 2]. 

White spot lesions (WSLs), defined as “white opacity,” occur because of subsurface enamel demineralization that is located on smooth surfaces of teeth. The reason for the white appearance is the changes in light-scattering optical properties of the decalcified enamel. Various risk factors such as acid-producing bacteria, fermentable carbohydrates, and many host factors, such as poor oral hygiene, low salivary volume, and a sugary diet, further contribute to the development of these incipient lesions [3]. One of the host risk factors is the fixed orthodontics appliances: namely, brackets, bands, wires, and other applications add up more plaque retention sites and thus make oral hygienic measures more difficult [4]. 

Conservative dentistry no longer favors the “drill and fill” concept and supports the reversal of lesions via remineralization [5]. Remineralization is the process of repairing minerals to the hydroxyapatite structure. It is three-dimensional, where the lost ions must be substituted with ions having the same shape, the same size, and the same electrical charge as those lost from the lattice [1]. White spot lesions could be reversed with fluoride as it enhances remineralization. Yet, clinically, fluoride did not reveal an obvious positive visible effect. Reviews by some researchers [6–8] showed that different forms, types, and concentrations of fluoride agents have little effectiveness to remineralize WSLs, as their availability to deliver net remineralization is limited by the availability of calcium and phosphate ions; hence, surface remineralization causes little aesthetic and functional improvement [9]. 

Regenerative medicine-based dental approaches, where the damaged tissues are substituted with biologically similar tissues, hence shift from reparative to regenerative dentistry [10]. A new approach to reverse and mask white spot lesions is the application of the self-assembling peptide P11-4.

On applying the P11-4-containing solution, P11-4 forms tapes and ribbons within seconds, and fibrils and fibers within the following 24 h under physicochemical conditions. These resulting self-assembling peptide fibers forming the 3D Self-Assembling Peptide Matrix (SAPM) can grow into a significant length of peptide diffusing into the lesion [11]. 

In the lesion, it is thought to self-assemble spontaneously and produce 3-dimensional gels comprised of self-styled β-sheet aggregates. In this manner, the attachment of calcium and phosphate from saliva is supposed to be improved, so-called the body’s saliva-driven-remineralization. eventually de novo hydroxyapatite formation [12]. 

Owing to the limited evidence-based data in the literature concerning the self-assembling peptide p11-4 in the management of white spot lesions, it was found that it will be goal-directed to assess the clinical performance of this recently introduced biomimetic material using a randomized controlled trial, to examine the null hypothesis that self-assembling peptide p11-4 will have the similar clinical performance as fluoride delivery systems in the management of white spot lesion after orthodontic treatment.

## Materials and methods

### Trial registration and ethical approval

The protocol of the current study was registered on clinical trials with a unique identification number (I.D. NCT03930927.). Ethical approval was obtained before the start of the study. The study was approved by the Research Ethics Committee (CREC), Faculty of Dentistry with an ethical approval number (19754).

### Sample size calculation

A study was planned to be of a continuous response variable from independent control and experimental subjects to evaluate the remineralization potential of self-assembling peptide P11-4 compared to fluoride vehicle material using laser fluorescence. In a previous study published in 2012 by Du et al. [5], the response within each subject group was normally distributed with a standard deviation of 4.86. If the true difference in the experimental and control means is 4, it was necessary to study 24 experimental subjects and 24 control subjects to be able to reject the null hypothesis that the population means of the experimental and control groups are equal with probability (power) 0.8. To account for losses during follow-up, this was increased to 29 white spot lesions in each group. The Type I error probability associated with this null hypothesis test is 0.05. PS power and sample version 3.1.2 for Windows were used to calculate the sample size.

### Study design

A randomized controlled clinical trial, with two parallel groups designed with a 1:1 allocation ratio. The participants were randomly assigned into two groups (*n* = 58) according to the remineralizing agent used, where (A) group represents participants who received fluoride varnish containing 5% sodium fluoride and tricalcium phosphate (Clinpro White Varnish, 3 M ESPE, Australia, New Zealand), while the second group (B) represents participants who received self-assembling peptide (Curodont repair™, Credentis AG, Windisch, Switzerland).

The examination was performed under direct illumination using a dental chair light after drying the teeth with compressed air for 5 s. All the participants were examined by the same examiner (S. H.) to avoid intra-examiner errors. All patients were selected according to inclusion and exclusion criteria to have non-cavitated white spot lesions.

### Eligibility criteria

## Patients eligible for the study complied with all the following:

- The patients’ age range between the age from 18 to 25 years old [5].
- Gender: males or females [5].
- Good oral hygiene with a plaque index score 0 or 1, good general health, and patient compliance [5].
- Active non-cavitated carious white spot lesions [13].
- Had completed fixed orthodontic treatment within the past 2 weeks [14].
- No systemic diseases or concomitant medication affects salivary flow in order not to negatively affect the remineralizing process [10].

## However, patients excluded from the study complied with:

- Participant in another trial [13].
- Patients with tetracycline pigmentation, dental fluorosis, or enamel hypoplasia to avoid any false-positive results [5, 15].
- Participants who had evidence of reduced salivary flow, systemic, or medical complications [13].
- Participants with cavitated lesions [5].

Eligible patients were recruited from the outpatient clinic of the Conservative Dentistry department in the Faculty of Dentistry, Cairo University, according to the participant timeline and signed informed consent.

### Random sequence generation (randomization)

Simple randomization was assigned for participants by generating numbers from 1 to 58 using Random Sequence Generator, Randomness and Integrity Services Ltd (http://www.random.org/). Each generated random number represented assigning the patient to intervention (B) or comparator group (A) in a random manner.

### Allocation-concealment mechanism

The allocation of remineralizing agents to groups was performed through an opaque sealed envelope to ensure complete concealment. Figure 8 shows the CONSORT 2010 flow diagram.

### Blinding

The patient and assessors were blinded to the material assignment while the operator did not, due to the difference in material presentation and its application protocol.

### Clinical examination of white spot lesions

Full dental and medical history for the patients was taken. A clinical examination of an active white spot lesion was performed to assess the color on the labial aspect of anterior teeth. WSLs defined as “white opacity” occur because of subsurface enamel demineralization that is located on smooth surfaces of teeth. These were assessed via dryness test by gentle drying for 5 s and via DIAGNOpen score [5].

### Material application

Clinpro White Varnish, 3 M ESPE, 5% sodium fluoride was applied under manufacturer instructions as follows: dryness of the affected tooth was unnecessary as it sets in presence of saliva. Then, a thin layer of varnish was applied with a brush in strokes. No rinsing, suction, or drying was required. The patient was instructed to avoid solid foods, brushing, and flossing for 4 h after application treatment; during this time, soft food and liquid might be consumed.

Curodont repair™ was applied according to the manufacturer’s instructions. Starting with the application of 2% NaOCl for 20 s to remove any plaque residual, then rinsed for 20 s and gently air-dried. The white spot lesion was etched with 35% phosphoric acid (Dental Technologies, Inc., USA) for 20 s, to open the pores to the subsurface lesion and then rinsed with water for 20 s and under moisture control [15]. Curodont repair ™ applicator unit was activated by pushing the two cylinders together. A white spot lesion was treated by gently pressing the tip directly onto the tooth surface and the product was left for 5 min to ensure its diffusion, till the tooth surface appears dry.

The remineralizing process of the white spot lesion was assessed quantitatively using the DIAGNOdent pen and qualitatively using the ICDAS scoring system according to time to the remineralizing agent used (T), where T0 represents the score taken before any treatment. T1 represents the score taken after 3 months follow-up and the T2 score represents the score taken after 6 months of follow-up.

Patients in both groups were advised not to brush or chew food for at least 4 h after treatment. Only soft food and drink could be consumed. The patients were asked to use a soft toothbrush and fluoridated toothpaste as an oral hygiene regimen.

### Statistical methods

This study was performed to compare the qualitative and quantitative effects of two remineralizing agents on post-orthodontic white spot lesions comprising different theories. The first remineralizing agent is the self-assembling peptide P11-4 which follows the non-classical theory of remineralization while the second one is the fluoride varnish material that follows the classical theory for the remineralization.

The mean and standard deviation values were calculated for each group in each test. Data were explored for normality using the Kolmogorov–Smirnov and Shapiro–Wilk tests, remineralizing process data showed parametric (normal) distribution, while ICDAS data showed non-parametric (not-normal) distribution.

An independent sample *t*-test was used to compare two groups in non-related samples for parametric data. To compare more than two groups in related samples, a repeated measure ANOVA was used. To compare two groups in related samples, a paired sample *t*-test was performed. The effect of interaction between multiple variables was tested using a two-way ANOVA.

The Mann–Whitney test was used to evaluate two groups in unrelated samples using non-parametric data. To compare more than two groups in related samples, the Friedman test was utilized. To compare two groups in related samples, the Wilcoxon test was utilized. The significance level was set at *p* ≤ 0.05. Statistical analysis was performed with IBM® SPSS® Statistics Version 20 for Windows.

## Results

### Demographic data

#### Gender and age

Regarding the gender distribution, 14 males (48.3%) and 15 females (51.7%) participated in Group A, while 11 males (37.9%) and 18 females (62.1%) participated in Group B. There was no statistically significant difference between the tested groups (*p* value = 0.430). The mean age value and standard deviation (SD) for Group A was 21.62 ± 2.78 with the age ranged between 18 and 25 years, while for Group B, it was 21.45 ± 2.53 with the age range between 18 and 25 years. There was no statistically significant difference in age between the tested groups (*p* value = 0.806), as shown in Table 1.

**Table 1:** The mean and standard deviation (SD) of age and frequency and percentage of gender for tested groups

Variables		Demographic data				
		(Group A)		(Group B)		*p* value
		Mean/*n*	SD/%	Mean/*n*	SD/%	
Age (years)		21.62	2.78	21.45	2.53	0.806 ns
Gender (*N*, %)	Females	15	51.7%	18	62.1%	0.430 ns
	Males	14	48.3%	11	37.9%	

*ns* non-significant (*p* > 0.05)

### Quantitative assessment of the remineralizing process using DIAGNOpen

#### Effect of time

##### Group A (fluoride varnish with tricalcium phosphate)

There was a statistically significant difference between T0 (baseline assessment before applying any remineralizing agent), T1 (after applying the remineralizing agents by 3 months), and T2 (after applying the remineralizing agents by 6 months) groups where *p* < 0.001. A statistically significant difference was found between T0 and each of the T1 and T2 groups where *p* < 0.001. Also, a statistically significant difference was found between T1 and T2 groups where *p* < 0.001. where the highest remineralizing potential is represented in T2 with a mean of 10.51 and standard deviation of 1.94, followed by T1 with a mean of 12.19 and standard deviation of 1.91, followed by T0 before applying any remineralizing agents with a mean of 16.46 and standard deviation of 2.91 (Table 2).

**Table 2:** The mean and standard deviation (SD) values of remineralizing evaluation along different periods of different groups

Variables	Remineralizing process using DIAGNOdent pen				
	Group A (fluoride varnish)		Group B (SAP)		*p* value
	Mean	SD	Mean	SD	
T0	16.46	2.91	14.29	3.97	0.050 ns
T1	12.19	1.91	8.90	2.82	< 0.001*
T2	10.51	1.94	6.45	1.99	< 0.001*
*p* value	< 0.001*		< 0.001*		

^*^Significant (*p* < 0.05); *ns* non-significant (*p* > 0.05)

##### Group B (biomimetic self-assembling peptides):

There was a statistically significant difference between T0, T1, and T2 groups where *p* < 0.001. A statistically significant difference was found between T0 and each of the T1 and T2 groups where *p* < 0.001. Also, a statistically significant difference was found between T1 and T2 groups where *p* < 0.001, where the highest remineralizing potential is represented in T2 with a mean of 6.45 and standard deviation of 1.99, followed by T1 with a mean of 8.90 and standard deviation of 2.82, followed by T0 before applying any remineralizing agents with a mean of 14.29 and standard deviation of 3.97 (Table 2).

#### Effect of groups

##### Baseline assessment before applying the remineralizing agents (T0)

There was no statistically significant difference between Group A (fluoride varnish) and Group B (self-assembling peptides) where *p* = 0.050. The highest mean value of 16.46 was found in Group A, while the lowest mean value of 14.29 was found in Group B (Table 2).

##### Assessment after the usage of the remineralizing agent by 3 months (T1)

There was a statistically significant difference between Group A (fluoride varnish) and Group B (self-assembling peptides) where *p* < 0.001. The highest mean value of 12.19 was found in Group A, while the lowest mean value of 8.90 was found in Group B (Table 2).

##### Assessment after the usage of the remineralizing agent by 6 months (T2)

There was a statistically significant difference between Group A (fluoride varnish) and Group B (self-assembling peptides) where *p* < 0.001. The highest mean value of 10.51 was found in Group A, while the lowest mean value of 6.45 was found in Group B (Table 2).

### Qualitative assessment of the remineralizing process using ICDAS scoring system

The frequencies of ICDAS along with different time intervals of different groups are shown in Table 3).

**Table 3:** The frequencies of ICDAS along with different time intervals of different groups

Variables		ICDAS				
		Group A		Group B		*p* value
		*n*	%	*N*	%	
T0	**Score 0**	0	0%	0	0%	0.064 ns
	**Score 1**	2	6.9%	0	0%	
	**Score 2**	27	93.1%	29	100%	
	**Score 3**	0	0%	0	0%	
	**Score 4**	0	0%	0	0%	
	**Score 5**	0	0%	0	0%	
	**Score 6**	0	0%	0	0%	
T1	**Score 0**	0	0%	0	0%	0.087 ns
	**Score 1**	16	55.2%	10	34.5%	
	**Score 2**	13	44.8%	19	65.5%	
	**Score 3**	0	0%	0	0%	
	**Score 4**	0	0%	0	0%	
	**Score 5**	0	0%	0	0%	
	**Score 6**	0	0%	0	0%	
T2	**Score 0**	0	0%	1	3.4%	0.277 ns
	**Score 1**	19	65.5%	19	65.5%	
	**Score 2**	10	34.5%	9	31%	
	**Score 3**	0	0%	0	0%	
	**Score 4**	0	0%	0	0%	
	**Score 5**	0	0%	0	0%	
	**Score 6**	0	0%	0	0%	
*p* value		< 0.001*		< 0.001*		

#### Effect of time

##### Group A (fluoride varnish with tricalcium phosphate)

There was a statistically significant difference between T0 (baseline assessment before applying any remineralizing agent), T1 (after applying the remineralizing agents by 3 months), and T2 (after applying the remineralizing agents after 6 months) groups where *p* = 0.001. A statistically significant difference was found between T0 and each of the T1 and T2 groups where *p* = 0.005 and *p* = 0.003 respectively. No statistically significant difference was found between T3 and T6 groups where *p* = 0.564.

##### Group B (biomimetic self-assembling peptides)

There was a statistically significant difference between T0 (baseline assessment before applying any remineralizing agent), T1 (after applying the remineralizing agents by 3 months), and T2 (after applying the remineralizing agents after 6 months) groups where *p* < 0.001. A statistically significant difference was found between T0 and each of the T1 and T2 groups where *p* = 0.005 and *p* < 0.001 respectively. Also, a statistically significant difference was found between T1 and T2 groups where *p* < 0.001.

#### Effect of groups

##### Baseline assessment before applying the remineralizing agents (T0)

There was no statistically significant difference between Group A and Group B groups where *p* = 0.064.

##### Assessment after usage of the remineralizing agent by 3 months (T1)

There was no statistically significant difference between Group A and Group B groups where *p* = 0.087.

##### Assessment after usage of the remineralizing agent by 6 months (T2)

There was no statistically significant difference between Group A and Group B groups where *p* = 0.277.

### Correlation between the quantitative and qualitative analysis of the remineralizing process using DIAGNOdent pen and ICDAS:

As presented in Table 4 and Fig. 6, there was a moderate positive relationship between remineralizing process and ICDAS, *r* = 0.549, *p* (2-tailed) < 0.001.

**Table 4:** Correlation between remineralizing process using DIAGNOpen and ICDAS

Variables	Pearson correlation	
Remineralizing process using DIAGNOpen and ICDAS	Correlation coefficient	0.549
	Sig. (2-tailed)	< 0.001

### Representative photos illustrating the qualitative analysis using ICDAS

**Fig. 1:**
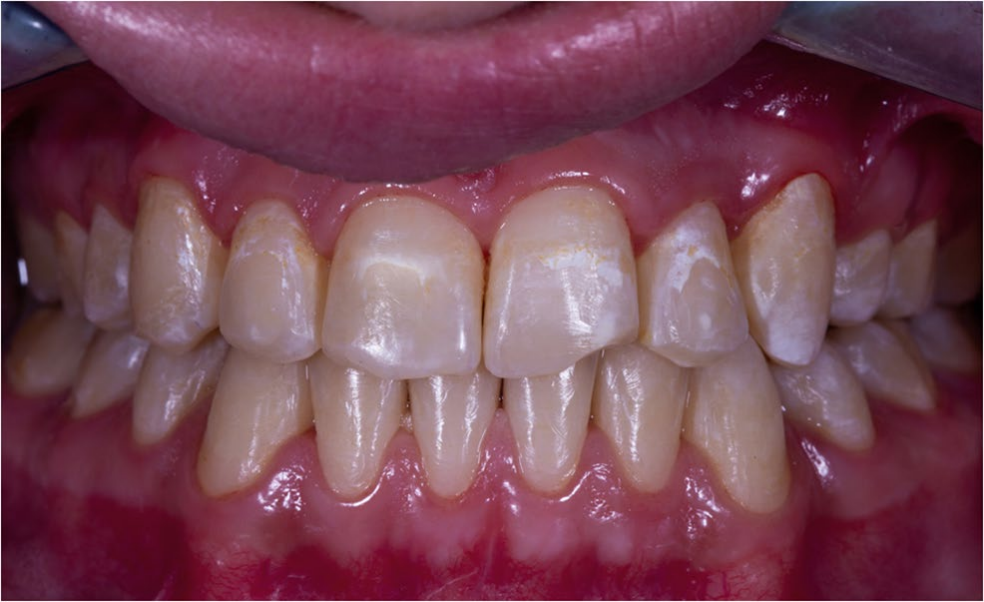
The white lesion presented around the brackets

A preoperative photo shows a post-orthodontics white spot lesion spread along the surface of the left central incisors. It appeared as a line around the orthodontic bracket in the right central incisors. Both are located mainly in the cervical third of the labial surface of the incisors. The same description applies to the lateral incisors where the white lesion presented around the brackets; however, it took a more circular in the left lateral one. Taking ICDAS score 2 as it appears in both wet and dry conditions, as shown in Fig. 1.

**Fig. 2:**
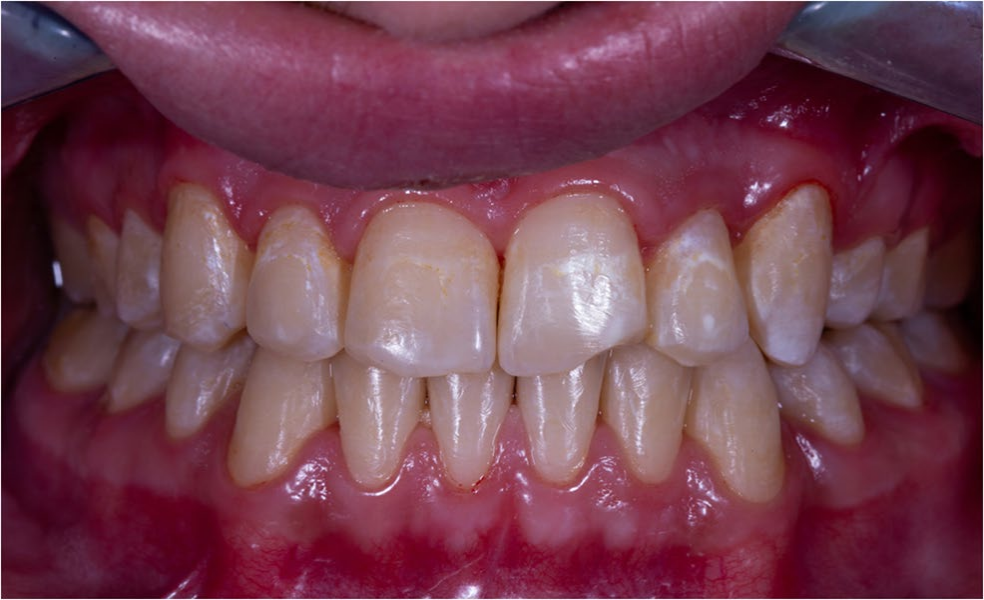
The post-orthodontic white spot lesions began to fade and become less intense

After 3 months of follow-up, the post-orthodontic white spot lesions began to fade and become less intense. They did, however, receive an ICDAS score of 2 because they appear in both wet and dry conditions as shown in Fig. 2.

**Fig. 3:**
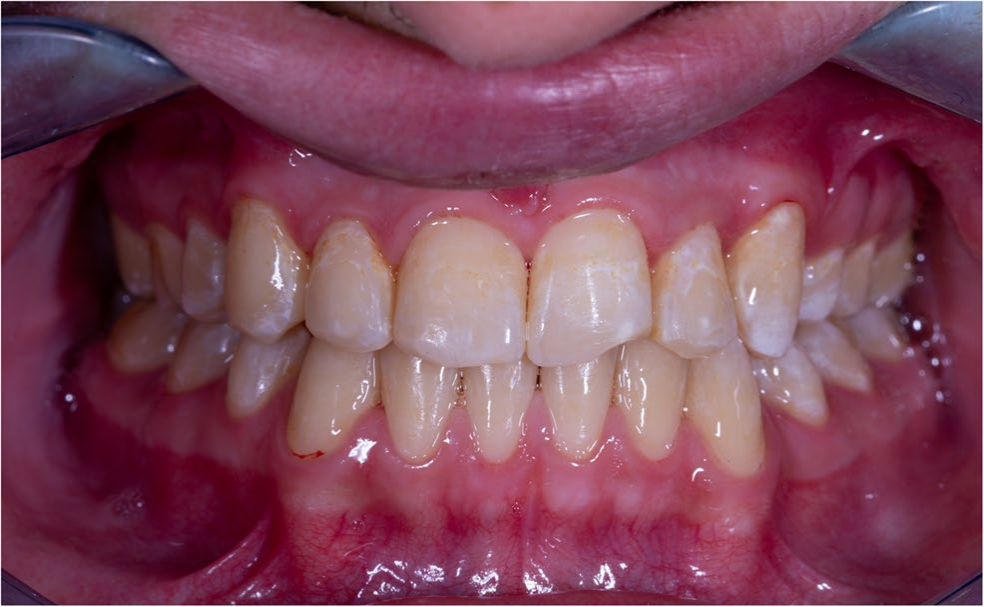
The rest of the white spot lesions turn out to be less severe and on their way to normal color

Six months later and adhering to the oral hygiene instructions, the white lesions began to fade. Keeping an eye on the right central incisor, where the white line vanished in the wet condition, and assigning an ICDAS score of 1. The rest of the white spot lesions turn out to be less severe and on their way to normal color, as shown in Fig. 3

**Fig. 4:**
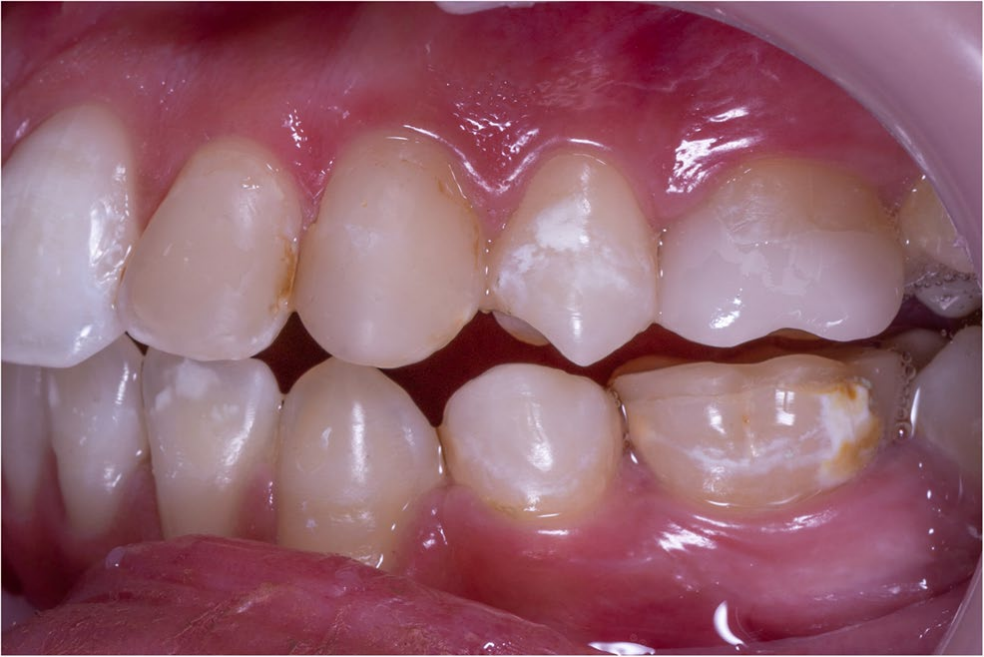
ICDAS score 2 for a prominent white spot non-cavitated lesion on the buccal surface of the upper left premolar that is visible in both wet and dry conditions

**Fig. 5:**
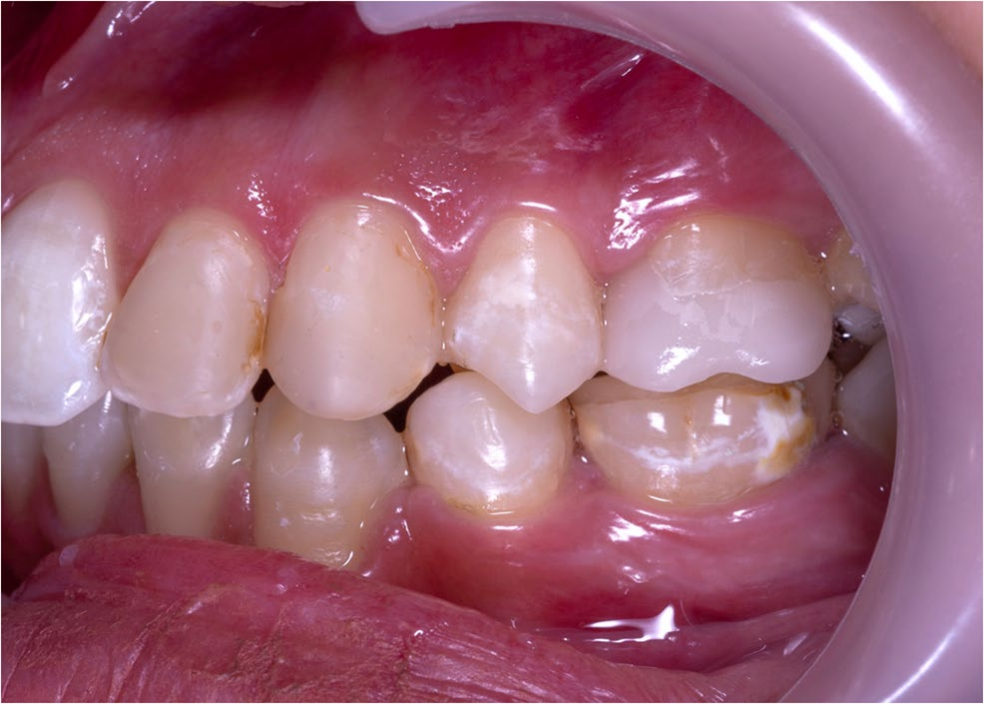
The lesions shrank in size and color after 3 months

**Fig. 6:**
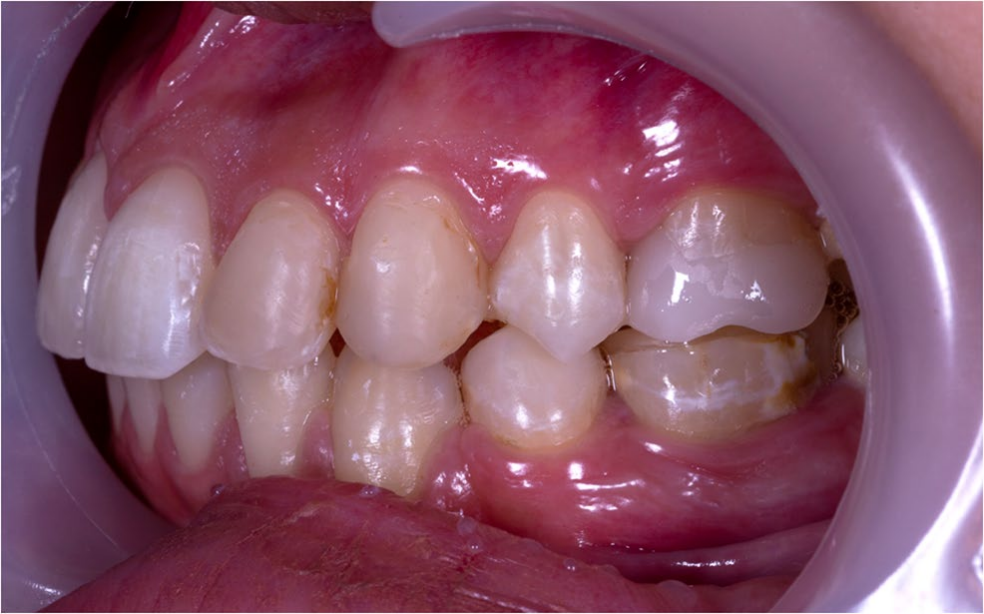
Lesions vanished after 6 months

**Fig. 7:**
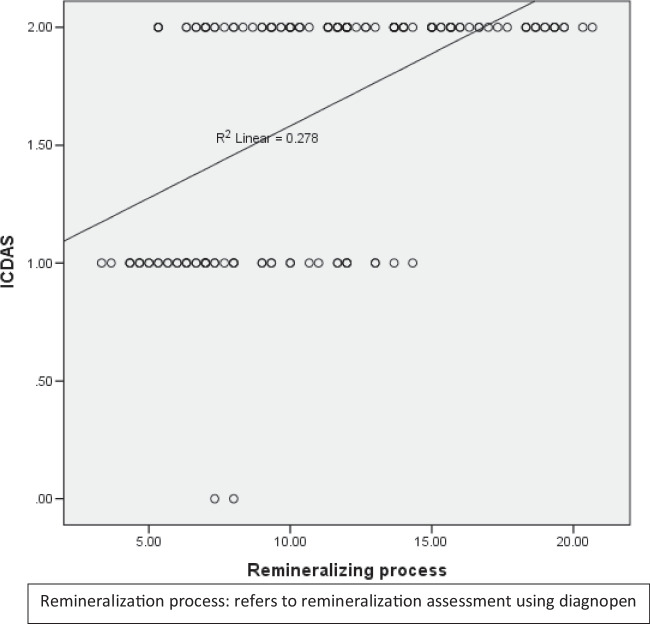
Scatter plot representing the correlation

**Fig. 8:**
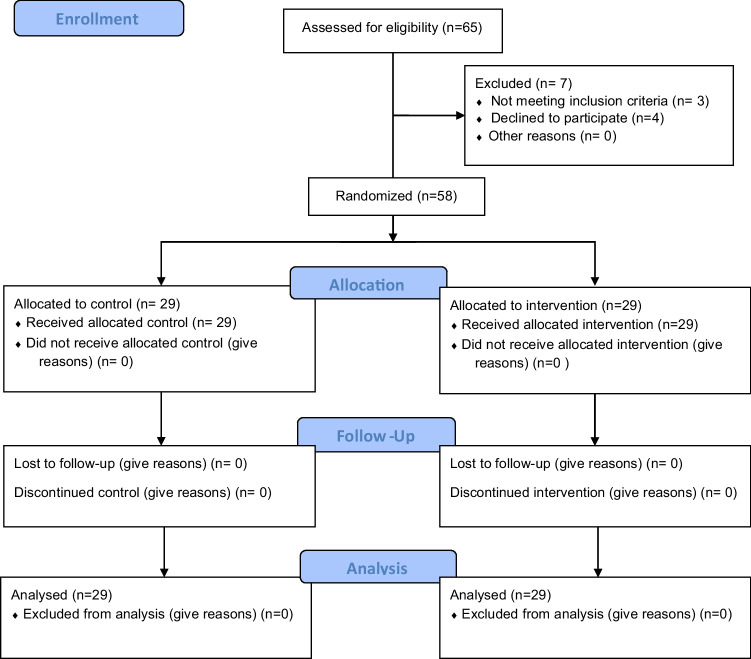
CONSORT 2010 flow diagram

ICDAS score 2 for a prominent white spot non-cavitated lesion on the buccal surface of the upper left premolar that is visible in both wet and dry conditions (Fig. 4). During the course of the follow-up, the severity of the lesion began to fade. After 3 months, the lesions shrank in size and color, as shown in Fig. 5, until they vanished after 6 months, as shown in Figs. 6, 7 and 8 with a score of 0.

## Discussion

Orthodontic therapy with fixed appliances may lead to a rapid increase in the volume of dental plaque with an elevated level of acidogenic bacteria. The low pH of dental plaque and subsequent imbalance of the remineralization-demineralization equilibrium favor demineralization in areas where optimum oral hygiene becomes difficult [5]. 

Clinically, enamel carious lesions in their early stage look like white, opaque spots softer than the surrounding sound enamel, and their whiteness increases with air drying. The early carious enamel lesion is characterized by subsurface damage with an intact enamel surface. This progressive subsurface demineralization, if not reversed, will lead to cavitation and eventually needs to be restored. Moreover, natural remineralization is difficult to be attained due to low calcium, phosphate, and fluoride concentration in the saliva. That is why intervention using remineralizing agents is needed to promote the mechanism of ion exchange to reverse the lesion avoiding invasive interventions [16, 17]. 

Tooth enamel regeneration is the goal today, through the true regeneration of hydroxyapatite crystals in subsurface lesions. This in fact can be mimiced by guided enamel repair with the use of self-assembling peptide P11-4 (SAP11-4) where monomeric P11-4 diffuses through the pores of decalcified enamel, fiber formation is triggered, and the 3D matrix is produced [11]. Primarily, the small protein molecules self-assemble to form a 3D scaffold in the subsurface lesion, mimicking the protein that is laid by ameloblasts during tooth formation. This 3D scaffold has a great affinity to minerals. Then, the precipitation of calcium and phosphate from saliva takes place on the formed scaffold indicating subsurface remineralization in a bottom-up direction, confirming full-depth remineralization and total repair of the lesion, hence non-classical crystallization theory or particle-based crystallization theory [17]. 

Kirkham J (2007) was the first to publish the proof of concept that self-assembling peptide P11-4 facilitates biomimetic enamel remineralization [12]. These peptides act as a building block that can diffuse into the subsurface of carious lesions, forming 3D biomatrix scaffolds in the form of fibers, that contain clusters of negative charges—acting as calcium-binding sites—that guide the regeneration of dental enamel. In other terms, SAP11-4 has a high affinity toward calcium and phosphate with the potential to nucleate de novo hydroxyapatite crystals. Those calcium and phosphate ions are supplied from saliva, thus supporting the concept of the natural remineralization mechanism driven by saliva [16, 18].

In the current study, both quantitative and qualitative analyses using DIAGNOpen and ICDAS are used in the current study as none of the diagnostic methods alone is enough for the diagnosis of dental caries. DIAGNOdent device was evidenced to be an efficient addition to other detection methods in caries detection as stated by a systematic review in 2021 [19]. 

The qualitative analysis using ICDAS is considered to be an easy chairside system that omits the use of sharp explorer that may endanger the enamel during caries detection, so it encourages preventative strategies that enable remineralization of non-cavitated lesions [20]. On the other hand, it has a drawback of over- and under-diagnosis. This has paved the way for quantitative detection methods**.** Thus, it was beneficial to record the ICDAS scoring system with some representative photos so it could be able to notice the changes in white spot lesions appearance that will help to monitor the remineralization process and to adopt the remineralization technique [21]. 

The quantitative analysis utilizing DIAGNOpen can detect the initial carious lesion where the laser beam fluorescence of demineralized enamel is lower than that of normal sound enamel. Six hundred fifty nanometer red diode laser beam is applied to the surface of the tooth. It is collected using a single optical fiber, filtered by high-frequency light wavelengths, and calculated by a photodiode. The amount of low-frequency fluorescence that passes through the caries lesion is measured and quantified [22]. The bacterial products such as porphyrins get caught in areas of porosity on the surface. When all overlying dental plaque biofilm is removed by professional cleaning, the retained bacterial products can still be detected. When DIAGNOdent readings curve upwards over time, this is strongly associated with white spot lesions [23]. As a result of the measurement scale, the term “quantifiable laser fluorescence” is used. When fluorescence increases, so does the possibility of decay. Therefore, it has good sensitivity, specificity, and reliability. Moreover, an extra advantage of the DIAGNOdent pen over the traditional DIAGNOdent being cordless, handy, simple, and precise [21]. However, the most significant disadvantage of this device is that it can produce false-positive results when plaque and calculus are present. So it is critical to clean the tooth surface before using DIAGNOdent [24].

In the study, both ICDAS and DIAGNOpen showed a significant increase in remineralization of post-orthodontic white spot lesions in both groups and over different time intervals. However, the remineralizing capacity of self-assembling peptides was superior to that of the fluoride varnish.

Fluoride follows the concept of ion-to-ion attachment for eventual enamel crystal growth. It is also called **ion-based crystallization theory**. Ions function as building units or nuclei for crystal aggregation then cluster formation and eventually crystal growth [17]. One of the shortcomings of this concept is the inability to guide the formation of hydroxyapatite crystals to have ordered mineral structural design and architecture under physiological conditions [25]. 

Fluoride varnish presents a successful non-invasive treatment modality for reversing the lesion in its early stage as it contains high concentrations of fluoride in comparison to the daily used toothpaste and mouthwash. So, surface fluoridation is maintained owing to the high level of fluoride ions. Moreover, it was suggested by some researchers that the combined formulation of fluoride varnish with tricalcium phosphate (TCP) with its mild acidity affects the saliva buffering capacity and plaque pH whereas the baseline salivary pH after immediate application of the fluoride varnish with tricalcium phosphate (TCP) is 6.7 that reaches 6.9 after 12 weeks [26]. This almost neutral pH would be able to enhance the remineralizing potentiality of fluoride-based material, opposite to other in vitro studies [27] that speculate the necessity of acidic formulation of a fluoride varnish to form the CaF_2_-like precipitate. However, the required acidic environment might be obtained from the acidic salivary and plaque pH and not from the chemical formulation of the varnish. Ultimately, it is considered to be safe, feasible in the application, and capable of reversing the lesion [5]. This is revealed by the significant increase in the remineralizing potential of fluoride-based material on white spot lesions quantitatively by DIAGNOpen where *p* < 0.001 and quantitatively by the ICDAS scoring system where *p* = 0.001 over a time interval of 3 and 6 months. This is following Kobeissi et al. [20] whose results showed a significant decrease in DIAGNOpen reading and ICDAS codes over time where *p* value < 0.001 that supports the mechanism of action of fluoride that acts by enhancing remineralization through ion deposition and suppressing the demineralization. Compatible results with Salamara et al. [28] revealed that 62% of white spot lesions were reversed taking a 0 score when the lesions were assessed through photographs using the ICDAS scoring system over a period of 16 weeks. Moreover, the current study qualitatively is following Singh et al. [29] as there was a significant decrease in the white spot lesions visually along with the 6 months follow-up where *p* < 0.01 but it conflicted with the same study quantitatively utilizing the DIAGNOdent, as there was not a statistically significant difference in DIAGNOdent scores where *p* > 0.05. Besides, it disagreed with Huang et al. [30] who reported that fluoride varnish did not add any beneficial value over the regular home care regimen over a period of 8 weeks.

Perhaps, it is attributed to the use of fluoride varnish that contains 5% sodium fluoride and tricalcium phosphate. During the manufacturing process, there is a protective barrier around the calcium ions. Once the varnish becomes in contact with the enamel surface and saliva, this barrier is broken, making fluoride, phosphate, and calcium ions enhance ion remineralization [20].

The remineralizing process of self-assembling peptide P11-4 showed significantly superior ability in enamel repair when compared to fluoride-based material. There was a statistically significant difference between Group A (fluoride vehicle material) and Group B (self-assembling peptides) groups where *p* < 0.001. The highest mean value of 12.19 was found in Group A, while the least mean value of 8.90 was found in Group B after a 3-month follow-up period. However, there was a statistically significant difference between the two groups where *p* < 0.001. The highest mean value of 10.51 was found in Group A, while the least mean value of 6.45 was found in Group B after a 6-month follow-up. This was ascribed to the ability of SAP to penetrate the subsurface lesion and build newly hydroxyapatite crystals from bottom to top, in contrast to fluoride, which remineralizes the enamel surface and cannot remineralize past the surface zone [17]. 

Brunton et al. [13] conducted a clinical study that is considered to be the first-in-man clinical safety trial to detect the safety and efficacy of self-assembling peptides on class V white spot lesions. A single application of P11-4 on early carious lesions led to a significant reduction of the lesion size within 30 days, as assessed on clinical photographs [13].

In post-orthodontic white spot lesions, self-assembling peptides P-114 displayed greater remineralization properties when mineral content was measured through radiographic and digital subtraction radiography, as in the case series by Schlee M [15], where 20 out of 28 patients revealed partial to complete regression of the lesion. That was in agreement with other researchers [31] who stated lower laser fluorescence values with self-assembling Peptide P11-4 Matrix (Curodont Protect) in comparison to the fluoride control group, in an in situ clinical trial [26]. There was a statistically significant difference between the two groups where *p* < 0.0001. The test group with P11-4 showed significantly more remineralization than the positive control with fluoride (*p* = 0.003). The authors stated that P11-4 is capable of preventing and remineralizing the dental enamel around orthodontic brackets.

Welk et al. (2020) documented a noticeable decrease in impedance readings (using CarieScan) after self-assembling peptide P11-4 application. The mean impedance value of the self-assembling peptides group showed 46.7 that decreased to 19.7 after 180 days. Also, the lesion size decreased from 8.8 to 6.5 after 180 days of SAP application [18].

As for the qualitative analysis using ICDAS, there is harmony between the results of the current study and the clinical study that was verified by other researchers [3] who measured early enamel lesion changes visually, via ICDAS II scoring. SAP showed a statistically significant improvement in reversing the lesion, over fluoride after 3 months of application. This was highlighted when some lesions had reverted from ICDAS scores 2 and 3 to an ICDAS score 0 upon SAP application, while no lesions exhibited reversal below ICDAS score 1 in the fluoride group. This was in accordance with another study [32] which stated that both self-assembling peptide P11-4 and fluoride showed a gradual decrease in the ICDAS score from baseline, reaching the lowest score after 6 months. Self-assembling peptide P11-4 (SAP) showed greater lesion regression than fluoride. Correspondingly, some authors [33] measured the morphometric changes by assessing photographs of the early buccal carious lesion after application of self-assembling peptide P11-4 and fluoride varnish after 30, 90, 180, and 360 days. The investigators reported lesion regression and reversal in the SAP group while the fluoride group remained stable.

In the present clinical trial, there was a moderate positive relationship between quantitative analysis through the remineralizing process and visual or qualitative assessment using the ICDAS scoring system. This may be contributed to the occurrence of remineralization whether through the classical theory or the non-classical theory by decreasing in laser fluorescence. Also, it was revealed visually by fading the lesions. This agreed with other researchers [34] who used both quantitative (laser fluorescence) and qualitative (ICDAS-II codes and Nyvad criteria) analysis to estimate the effect of SAP when used in combination with fluoride varnish or twice-weekly application of polymeric self-assembling peptide matrix (Curodont Protect). Laser fluorescence showed superior results over fluoride varnish alone where *p* < 0.0005. Moreover, ICDAS codes showed partial regression for both groups 6.7% and 20% while in the fluoride varnish, it showed partial regression by 23.3%. As for Nyvad criteria, it revealed superior caries inactivation for the combined therapy over the fluoride varnish alone. This agreed with Alkilzy et al. [35] who stated that there is a statistically significant from SAP in combination with fluoride, over the use of fluoride alone, when measured using ICDAS-II scoring and Nyvad criteria. Moreover, the added proof was provided in this study via quantitative analysis, where there was a statistically significant decrease in laser fluorescence readings in the test group (combination of SAP and fluoride), in comparison to the control group (fluoride varnish only) at 3 and 6 months of follow-up when measured using DIAGNOdent.

All these findings support the repair potential of the self-assembling peptide that can perform a true stable 3D scaffold. Thanks to the surface pre-treatment through conditioning the surface followed by surface etching, which allows high surface reactivity for better peptides diffusion, then again negatively charged scaffold formation acting as binding sites for positively charged calcium and phosphate ions confirming subsurface remineralization in a bottom-up direction [11]. Conversely, SAP-14 is regarded not simply in the application as fluoride-based material. The multistep, careful application and cost are stated as the main drawbacks of the material [20]. That is why biomimetic remineralization is a double-edged weapon.

Eventually, enamel repair through biomimetic remineralization utilizing self-assembling peptides is considered to be a new avenue for remineralizing, reversing, and repair the early enamel lesion. Without a doubt, it will open the door to regenerative dentistry.

According to the previously discussed results of this study, the null hypothesis is rejected where self-assembling peptides have a superior remineralizing potential over fluoride-based delivery systems quantitatively using laser fluorescence; however, both materials have the same visual effect on masking the early lesion. This new approach possibly reverses and masks off post-orthodontics white spot lesions.

## Conclusions

Within the limitation of the present study, biomimetic remineralization promoted by self-assembling peptides has achieved successful subsurface remineralization making the material a promising guide to lesion regression in post-orthodontic therapy.

## Recommendations

1. Clinical trials with further long-term follow-up studies are recommended.
2. Further investigations are needed to assess self-assembling peptides on advanced and cavitated lesions.
3. The material’s efficacy in the management of dentin hypersensitivity needs to be investigated.
4. Investigating the material as a founder to the remaining dentin in deep carious lesions is also recommended.
5. Research studies are needed to evaluate the synergistic effect of self-assembling peptides with other agents based on ion crystallization theory.

## Supplementary Information

Below is the link to the electronic supplementary material.

Supplementary file1 (DOCX 14 KB)

Supplementary file2 (DOCX 1472 KB)
