# Supplementary material for: Evaluation of the remineralizing effect of biomimetic self-assembling peptides in post-orthodontic white spot lesions compared to fluoride-based delivery systems: randomized controlled trial
Source: Clin Oral Investig. 2022 Oct 26;27(2):613–24. doi: 10.1007/s00784-022-04757-7 (PMC9889428; doi:10.1007/s00784-022-04757-7)
Supplement: Supplementary file 1 — Supplementary file1 (DOCX 14 KB) [file 784_2022_4757_MOESM1_ESM.docx]

**Figure (1):** Pre-operative photo for the fluoride varnish group

**Figure (2):** 3 months follow up postoperative photo for the fluoride varnish group

**Figure (3):** Post-operative photos 6 months follow up for the fluoride varnish group

**Figure (4):** Pre-operative photo for the SAP-14 group

**Figure (5):** Post-operative photos 3 months follow up for the SAP-14 group

**Figure (6):** Post-operative photos, 6 months follow up for the SAP-14 group
